# Detection of *Chlamydia trachomatis* in Pap Smear Samples from South Khorasan Province of Iran 

**DOI:** 10.22074/ijfs.2018.5064

**Published:** 2018-01-15

**Authors:** Davod Javanmard, Mahmoodreza Behravan, Malaknaz Ghannadkafi, Alireza Salehabadi, Masood Ziaee, Mohammad Hasan Namaei

**Affiliations:** 1 Infectious Diseases Research Center, Birjand University of Medical Sciences, Birjand, Iran; 2 Faulty of Nursing and Midwifery, Birjand University of Medical Sciences, Birjand, Iran

**Keywords:** Cervical Cancer, *Chlamydia trachomatis*, Iran, Pap Smear, Sexually Transmitted Infection

## Abstract

**Background:**

*Chlamydia trachomatis* (CT), the most common bacterial sexually transmitted infection (STI), leads to pelvic inflammatory disease, infertility and chronic pelvic pain in women as well as an increased risk of vertical transmission, conjunctivitis and pneumonitis in infants. It may also be a co-factor along with human papillomavirus (HPV) in cervical cancer progression. We aimed to determine the prevalence of CT genotypes in genital specimens of women from South Khorasan, Iran and to test the association between CT and cytology statistics.

**Materials and Methods:**

This was a cross-sectional study on 248 Pap smear samples from women who visited a gynecologist for routine Pap smear testing in South Khorasan province. Nested polymerase chain reaction (PCR) was used to test the residual fluids of Pap smears for CT-DNA after cytological examination. Direct sequencing, alignment and phylogenic analyses were performed on eight samples to identify their genotypes.

**Results:**

The mean age of patients was 37.54 ± 5.21 years. Most samples had a normal cytology (214 cases, 86.29%). Overall, 31 samples were positive for CT infection (12.5%) of which 20 (9.34%) were normal and 11 (32.35%) were abnormal, with the frequency difference being significant (P=0.022). The co-infection of CT/HPV in total was identified in 14 cases (5.6%). The results of sequencing eight samples out of the 31 CT positive samples revealed the detection of genotypes D and E, each with four cases.

**Conclusion:**

We show that a high prevalence of genital CT infection is present in women with both normal and abnormal cytology; however, the higher prevalence among women in the abnormal group may indicate its involvement in cervical neoplasia.

## Introduction

*Chlamydia trachomatis* (CT) is the most common sexually transmitted bacterial infection (1). Chlamydia species are aerobic obligate intracellular bacteria with a gramnegative cell wall. Because of their inability to produce ATP, they are dependent on their host energy (2). The major outer membrane protein (MOMP), a principle component of the CT cell wall, is encoded by the *omp1* gene, which includes four variable domains (VD) interspersed among 5 conserved domains (3). Based on minor variation in three VDs, which are exposed on the surface of the membrane, CT currently has 19 genotypes (A to K, L1 to L3, Ba, Da, Ia and L2a) (4), among which genotypes D to K are urogenital pathogens and responsible for neonatal conjunctivitis, genotypes A, B and C are related to trachoma, and L1 to L3 are responsible for the sexually transmitted infection, lymphogranuloma venereum (5).

Chlamydial infection in women can cause urogenital inflammations including urethritis, cervicitis and salpingitis (6). Also, infants born from mothers with active CT infection may develop conjunctivitis or pneumonia (2). Unfortunately, most CT infections are asymptomatic (70% in women) (7). This is challenging for early detection and treatment, and thus increases transmission. Risk factors that can be attributed to this infection are age (those aged 15-24 are most affected) and gender (women are more prone to infection than men) (8).

The co-infection of CT with other sexually transmitted pathogens may have complicated consequences. Genital CT infection may increase human immunodeficiency virus (HIV) viral shedding, therefore, identifying and treating patients with CT infection may reduce the genital transmission of HIV (9). Several studies have reported the coexistence of CT in cervical intraepithelial neoplasia (CIN) induced by human papillomavirus (HPV) infection (10). There are some reports of a higher prevalence of CT in HPV-positive populations (11), yet CT has been introduced as an independent risk factor for developing CIN (12). 

There are several diagnostic methods for CT, such as isolation in cell lines, immunofluorescence, serologic assays and molecular testing methods such as polymerase chain reaction (PCR) (13). CT infection is easily treatable with accessible antibiotics, therefore, given the asymptomatic nature of most CT infections, the early detection of this sexually transmitted infection could enhance treatment and reduce the risk of a re-infection and/or transmission to others (9). 

Although CT infection has been proven to be the most prevalent sexually transmitted infection (STI) (1), there is still no clear information on its prevalence in South Khorasan province in eastern Iran. In addition, there is a lack of data on the co-infection of CT/HPV and the prevalence of CT among different cytology groups in association with cervical malignancies in Iran. We therefore aimed to address these by undertaking cytological and sequencing analyses. 

## Materials and Methods

This was a cross-sectional study performed in Birjand, South Khorasan province of Iran from May 2015 to October 2016. The age of women ranged from 17 to 45 years, all of which were referred for routine Pap smear test. Those who had taken antibiotics within 3 weeks prior to their visit were excluded from the study. All patients signed an informed consent. This work was approved by the Ethics Committee of the Vice-chancellor for Research of Birjand University of Medical Sciences (#1393-12-07). Data collection and recording were performed based on questionnaires and forms. Total endocervical epithelial cells were collected from 248 women visiting different gynecologists in Birjand. These samples had been previously checked for HPV-DNA and their results were used in this study (14).

### DNA extraction

On the same day of cytological examination, the residual fluids containing endocervical cells were processed for DNA isolation. A Bioneer DNA extraction kit (Bioneer Co, South Korea) was used according to the manufacturer’s instructions with minor alterations including the preheating of samples and an additional round of centrifugation. The extracted DNA was checked using a Nanodrop Biophotometer (Eppendorf D30, Germany) at 260/280 nm, and samples with a ratio between 1.8- 2 were selected. An internal control gene (*ß-globin*) was selected for amplification to confirm the process of cellular DNA extraction.

### Amplification of *ß-globin* and Omp1 gene

The isolated DNA was subjected to PCR using primers beta 1 (5.-TCAACCCTACAGTCACCCAT-3.) and beta 2 (5.-CTAACAATTACGAACAGCAATGAG- 3.), as previously described, to assess its integrity (15). Positive samples were then selected for CT testing with nested-PCR as previously described (16). Briefly, in the first round, 5 µl of extracted DNA was added to a reaction tube containing 25 pmol of each outer primer (NLO: 5.-ATGAAAAAACTCTTGAAATCG-3. and NRO: 5.-CTCAACTGTAACTGCGTATTT-3.), 0.2 mM of each dNTP, 1X PCR buffer, 2 mM MgCl_2_ and 2 U Taq DNA polymerase (Cinaclone, Iran) in a volume of 50 µl. The nested step was performed on 3 µl of the first-round PCR product as a template with inner primers NLI (5.-TTTGCCGCTTTGAGTTCTGCT-3.) and NRI (5.-CCGCAAGATTTTCTAGATTTC-3.) (16) under reaction conditions identical to the first PCR except that the concentration of MgCl_2_ was 1 mM. First- and second- round PCR reactions were performed using an Eppendorf thermocycler (Mastercycler Nexus, Eppendorf, Germany) under the following cycling conditions: 4 minutes preheating at 95ºC followed by 30 cycles of denaturation at 94ºC for 1 minutes, annealing at 57ºC for 1 minutes and extension at 72ºC for 1 minutes. A final extension step at 72ºC for 7 minutes was added to guarantee full-length products. The product of nested PCR was a 1050 base pair segment of the Omp1 gene that was visualized on a 1% agarose gel and stained with DNA Green Viewer.

### Genotyping

The products of nested PCR were sequenced bidirectionally using the same forward and reverse primers at Bioneer Company, South Korea (run on an ABI 3730XL DNA Analyzer). The obtained sequences were aligned using Mega BLAST to determine genotypes. The phylogenetic analysis in the given region of Omp1 was performed using MEGA6; the Jukes-Cantor model was selected for nucleotide substitution with Gamma distributed rates among sites. Selected codon positions were 1^st^, 2^nd^, 3^rd^ and noncoding sites. To assess the reliability of the phylogeny, 1,000 bootstrap replications were performed. The accession numbers of reference sequences of CT genotypes used in this study were KM369934 (E), X62918 (D), KM369939 (G), DQ064292 (J), KM369936 (F), AF202456 (Ia), DQ064282 (B), DQ064295 (L2), AF063204 (K), X16007 (H), FM872306 (A) and CP006945 (C).

### Statistical analysis

The type of distribution was checked, and skewness and kurtosis were in the range of (2, 2). The Chi-square test (or Fisher’s exact test when applicable) was used to test association and to compare between cytology and CT. The statistical significance was set at P<0.05. All statistical analyses were performed by Statistical Package for Social Sciences software version 17 (SPSS Inc, Chicago, IL, USA).

## Results

### Demographic population-based data

The demographic and clinical data of the 248 women screened are shown in Table 1. The mean age of patients was 37.54 ± 5.21 years. Most participants had a normal cytology (214 cases; 86.3%), however, 34 cases (13.7%) had an abnormal cytology result. In the abnormal group, there were 20 cases (58.82%) with atypical squamous cell of undetermined significance (ASCUS) and 14 (41.17%) with low-grade squamous intraepithelial lesions (LSIL). There were no cases with high-grade squamous intraepithelial lesions (HSIL) and/or cervical neoplasia. Based on cytological examination, observation of inflammatory cells and clinical data, 38 of all cases (15.32%) were found to have cervicitis.

### Prevalence of *Chlamydia trachomatis* among Pap smear samples

**Fig.1:**
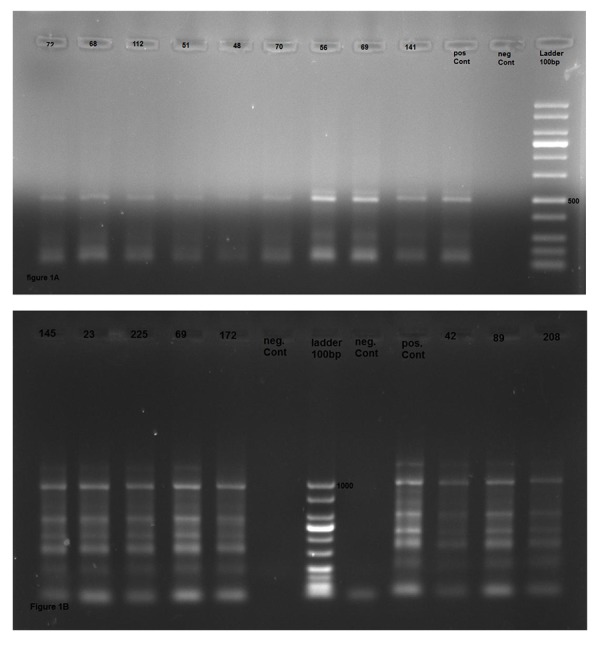
Agarose gel electrophoresis of polymerase chain reaction (PCR) products. A. The positive samples for amplification of human *ß-globin* gene revealed a 500 bp fragment band, and B. Positive samples for Chlamydia trachomatis (CT) have a 1052 bp product.

The results of PCR for the *beta-globin* gene demonstrated its amplification in all samples after agarose gel electrophoresis (Fig .1A). Based on PCR results, 31 cases (12.5%) were positive for CT (Fig .1B). The prevalence of CT among different groups is shown in Table 1. Among the samples with evidence of cervicitis, seven (18.42%) were positive for CT. The mean age of patients with CT infection was 36 ± 5.52 years. The distribution of CT in different age ranges is shown in Table 2. The modal age range was 21-30 years (130; 52.42%). The prevalence of CT and cervicitis was however higher in the first age range (18.18 and 27.27% respectively), and declined with age. 

### Genotyping of *Chlamydia trachomatis*

**Fig.2:**
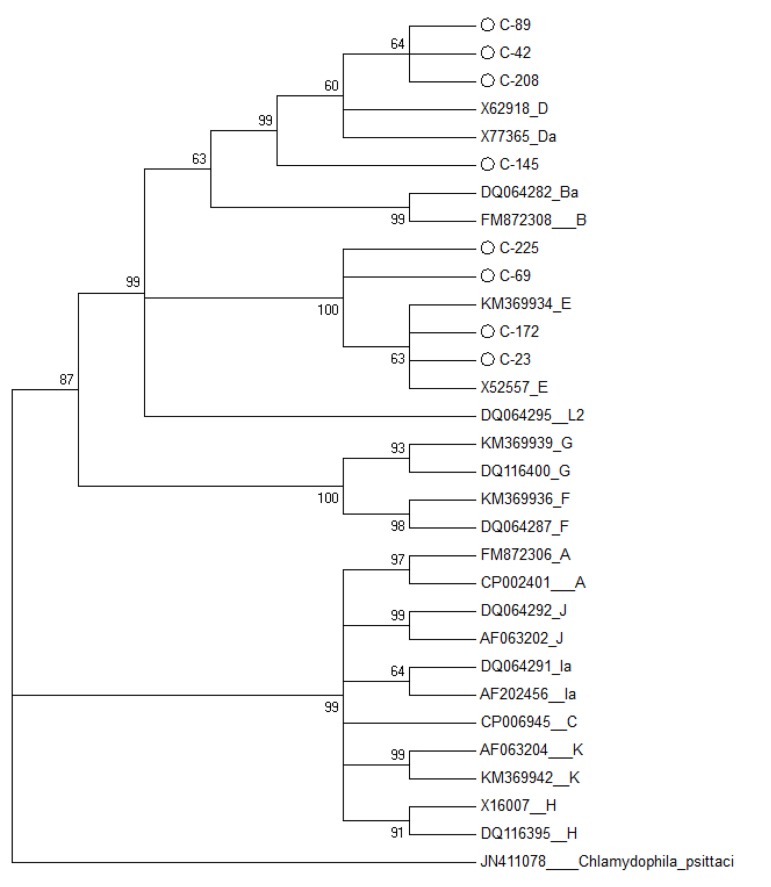
The phylogenic tree constructed based on the maximum likelihood method. Accession numbers and genotypes were given, and those in circle shape are the sequences reported here. *Chlamydia trachomatis* (CT) genotype D (X62918) was used as reference sequence and the *omp1* sequence of C. Pesittaci was used to root the tree.

The forward and reverse sequences obtained from eight samples were assembled to a consensus using CLC software (CLC Genomics Workbench 7, https://www.qiagenbioinformatics. com/), trimmed in Bioedit software, and subsequently submitted to NCBI under accession numbers KY468517 to KY468523. In search for homology via BLAST, half of samples belonged to genotype D and the other half belonged to genotype E (Fig .2).

Demographic and clinical characteristics of these genotypes are in Table 3. The cases positive for genotype D were younger (28.45 ± 3.26 years), although it was not statistically significant. Interestingly, in the cases positive for genotype E, the co-infection of HPV and LISL were found more frequently.

In isolates of genotype E, there were no mutations at the amino acid level; however, there was a missense mutation in a case with genotype D (i.e. thr326ala). There were also two silent mutations, C915T in two cases of genotype E and T956A in two cases, with genotypes D and E. Overall, the nucleotide region 900-1000, a part of the variable domain- .V, was more prone to have a mutation.

**Table 1:** The prevalence of CT among different cytological groups, as well as co-infection with HPV


Cytology	n (%)	Age (Y) Mean ± SD	HPV DNA n (%)	CT DNA n (%)	HPV/CT co-infection	P value

Normal cytology	214 (86.29)	35.55 ± 4.66	33 (15.42)	20 (9.34)	11 (5.14)	
Total abnormal	34 (13.7)	38.45 ± 4.21	12 (35.29)	11 (32.35)	3 (8.82)	0.022*
ASCUS	20 (58.82)	37.1 ± 3.35	8 (40)	5 (25)	2 (10)	
LSIL	14 (41.17)	35.3 ± 5.6	4 (28.57)	6 (42.85)	1 (7.14)	0.056**
Total	248	37.54 ± 5.21	45 (18.14)	31(12.5)	14 (5.64)	


CT; *Chlamydia trachomatis*, HPV; Human papillomavirus, ASCUS; Atypical squamous cell of undetermined significance, LSIL; Low-grade squamous intraepithelial lesions, *; The prevalence of CT was significantly different between total abnormal and normal cytology groups, and **; The prevalence of CT was higher in the LISL group than ASCUS.

**Table 2:** The prevalence of *Chlamydia trachomatis* according to age and cervicitis test result


Age ranges (Y)	Total number (%)	CT+/each group n (%)	CT+/total (%)	Cervicitis+/each group n (%)	CT+and cervicitis+/each group n (%)

≤20	11 (4.43)	2 (18.18)	0.8	3 (27.27)	1 (9.09)
21-30	130 (52.42)	16 (12.3)	6.45	20 (15.38)	4 (3.07)
31-40	81 (32.66)	9 (11.11)	3.62	12 (14.81)	2 (2.46)
40˃	26 (10.48)	4 (15.32)	1.6	3 (11.5)	0
Total	248	31 (12.5)	31 (12.5)	38 (15.32)	7 (2.82)


**Table 3:** Demographic and clinical characteristics of individuals with genotypes D and E identified in this study


Genotype	n	Mean age ± SD	Cervicitis n (%)	HPV n (%)	ASCUS n (%)	LISL n (%)

D	4	28.45 ± 3.26	2 (50)	1 (25)	3 (75)	0
E	4	34.51 ± 2.52	0	3 (75)	1 (25)	4 (100)
Total	8	31.48 ± 2.55	2 (25)	4 (50)	4 (50)	4 (50)


HPV; Human papillomavirus, ASCUS; Atypical squamous cell of undetermined significance, and LSIL; Low-grade squamous intraepithelial lesions.

## Discussion

The Pap smear test is approved for screening cervical abnormalities and is performed routinely around the world. Therefore, a large and continuous sampling is in progress and is accessible. This study showed the capacity of the liquid Pap smear to enhance the molecular detection of genital CT infection, as other studies have also indicated (17). This study was the first to assess the frequency of CT infection and genotypes of CT among women from South Khorasan, Iran. The observed frequency of CT in South Khorasan (12.25%) is comparable to other studies in Iran by Chamani-Tabriz et al. (18), Zahirnia et al. (19) and Eslami et al. (20) which reported the prevalence of CT as 12.6, 13.2, and 13.25% respectively. Other studies in Iran have shown the molecular detection rate of CT from 2.6 to 21.25% (21), and according to a meta-analysis, the pooled prevalence for genital CT in Iran was 12.3% (22).

The large variance observed in the reported data may be due to sampling size, sample source, experimental test, socio-economic state of the population and other factors. The CT prevalence has been reported at variable rates in other parts of the world such as 6.2% in Australia (23), and 1.1-10.6% in other countries (24). In this work, the prevalence of CT was higher among ages lower than 20 years (18.18%) and showed a decreasing pattern with age increase, albeit it was relatively high at ages of 40 years and more (15.32%). Other studies from Iran have also indicated a declining prevalence of CT proportional to senescence (25, 26), however, some studies have shown the highest frequency is in the 30-40 age groups (18, 27). Also, in other countries, there is a higher prevalence of CT in late teens and early youth (24). The early incidence of CT infection and its different age distribution may be due to physiologic changes of the vagina in addition to social behavior and lower marriage age, a frequent phenomenon in this province.

We found that the incidence of CT infection was higher among patients with abnormal cytology (32.35%). Interestingly, this figure was 42.85% for the LISL group, 25% for the ASCUS group 9.34% for the normal group, indicating an ascending pattern toward malignancy. In a case-control serological survey in Iran, a strong association between CIN and CT was identified. In specific, in the CIN and healthy group, there were 45 and 12.9% seropositive individuals respectively (28). Nonetheless, others have reported no significant association between CT and CIN (29, 30). An investigation in Argentina revealed a rising prevalence of CT from low levels in normal cytology (11%) to 47% in those with HSIL (11), which is consistent with our results.

This result confirmed the shared risk of CT and HPV infection in the development of cervical cancer. The co- infection rate of HPV and CT was 14/248 (5.64%) in the total sample set and 14/31 (45.16%) among CT-infected patients reported here. A study in Italy showed that 58% of CT-infected women were also positive for HPV (31), which is somewhat consistent with our results. Panatto et al. (32) reported this as 2.7% of total women, and Bianchi et al. (33) showed that 1.5% of girls younger than 20 were co-infected with CT/HPV. This result is consistent with a meta-analysis that demonstrated the association between CT and the risk of cervical cancer (34).

HPV and CT share similar transmission routes, and since CT may enhance the rate of other STI infections, it may have a role in the progression of cervical cancer. It may, however, be an independent co-risk factor of CIN with an unknown mechanism.

In the current study, the CT genotypes D and E were equally identified. These genotypes were also prominent in other genotyping surveys from Iran. Genotyping of CT from endocervical specimens in Shiraz identified genotype F (46.6%), E (33.3%) and D (13.3%) along with a singleton G (35). In a comprehensive genotyping study for genital CT in Ahvaz, genotype E was the most prevalent (31.5%), followed by F (23.1%), D (13%), K (9.2%), I (8.3%), G (7.5%), H (5.5%) and J (1.9%) (36). The lack of other genotypes in South Khorasan is interesting and shows a possible bottleneck effect. The insufficient number of samples genotyped may nevertheless have resulted in the absence of rarer genotypes.

## Conclusion

The results of this study revealed a relatively high prevalence of genital CT in East Iran and underscore the benefit of liquid Pap smear samples for molecular assays. The association between the rate of CT and CIN grade merits further investigation. Determining the prevalence and genotypes can provide important epidemiologic knowledge for transmission patterns, prevention, and treatment programs for controlling STI infections. Further investigations in this region are also needed to obtain a more reliable prevalence of CT and to determine its relevance to any other genital infections or cervical carcinoma.
